# Case Report: A rare case of unilateral renal clear cell carcinoma with synchronous contralateral ureteral metastasis

**DOI:** 10.3389/fonc.2025.1616814

**Published:** 2026-01-09

**Authors:** Yichen Qian, Yuanchen Lu, Qijie Zhang, Jianjun Xie, Hua Shen, Junpeng Deng

**Affiliations:** Department of Urology, The Affiliated Suzhou Hospital of Nanjing Medical University, Suzhou Municipal Hospital, Suzhou, China

**Keywords:** renal cell carcinoma, ureteral neoplasms, metastasis, nephroureterectomy, nephrectomy, biopsy

## Abstract

**Background:**

Renal cell carcinoma (RCC) rarely metastasizes to the contralateral ureter. Fewer than 10 such cases have been reported, mostly involving clear cell RCC (CCRCC) with associated hydronephrosis and renal insufficiency. The mechanism may involve retrograde tumor cell implantation via the urine. This report presents a rare case of unilateral CCRCC with synchronous contralateral ureteral metastasis and its treatment.

**Case presentation:**

A 77-year-old male patient presented with hematuria and right lumbar pain. Computed tomography (CT) imaging revealed a left renal mass and a small right ureteral mass with hydronephrosis and suggested malignancy in both masses. Owing to bilateral involvement and poor general condition, a staged approach was adopted. Laparoscopic partial nephrectomy of the left renal mass yielded clear cell renal cell carcinoma (CCRCC). Subsequent laparoscopic nephroureterectomy for the right ureteral tumor confirmed CCRCC metastasis. The patient recovered well and showed no metastasis at the fourteen-month follow-up.

**Conclusion:**

This type of metastasis deserves increased attention. However, there is currently no standardized treatment protocol for this rare condition. Our treatment involved firstly management of the renal tumor, followed by the contralateral ureteral tumor so that optimal safety and minimum oncological progression can be achieved.

## Introduction

The most common distant metastatic sites of renal cell carcinoma (RCC) are the lung, bone, lymph nodes, liver, adrenal gland and brain (1). Ureteral metastasis is relatively rare, especially contralateral ureteral metastasis, which occurs even less frequently in a synchronous manner. Fewer than ten cases of synchronous contralateral ureteral metastasis from RCC have been reported to date (2–7). Predominantly, these cases involve clear cell renal cell carcinoma (CCRCC), with hydronephrosis and renal insufficiency on the same side as ureteral metastasis. This condition may be associated with a poorer prognosis than metachronous metastasis due to its distinct phenotype (8). The mechanism for ureteral metastasis remains unclear. One proposed hypothesis is that tumor cells shed into the urine followed by retrograde implantation into the contralateral ureter (3). Here, we report a rare case of unilateral renal clear cell carcinoma with synchronous contralateral ureteral metastasis and our treatment.

## Case presentation

A 77-year-old male patient was admitted to our hospital due to hematuria on June 12, 2024, accompanied by mild lumbar soreness on the right and without any lower urinary tract symptoms or systemic symptoms. The patient had a history of hypertension and diabetes, both of which were managed with medication.

**Figure 1:**
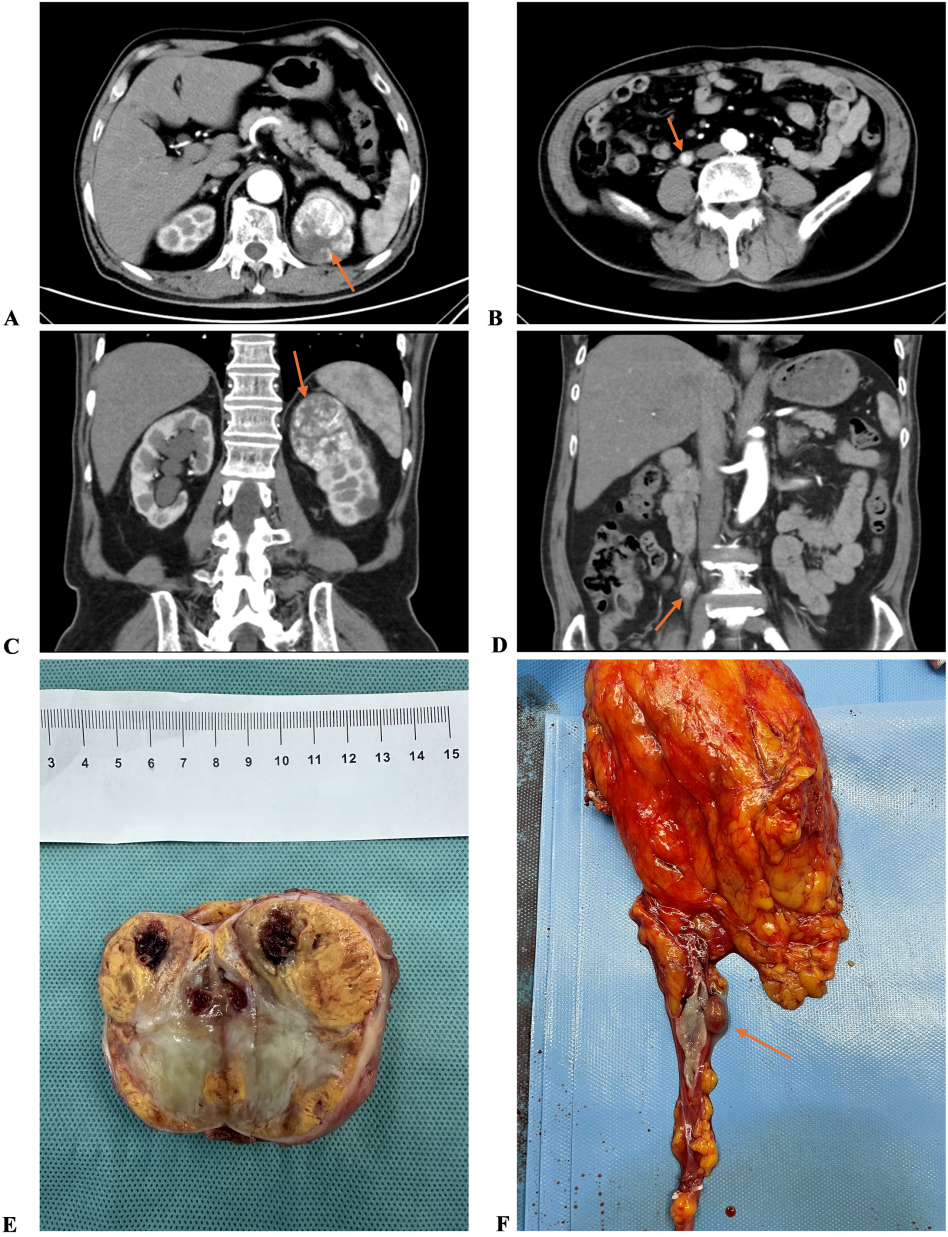
The CT images and gross specimens of the renal and ureteral tumors. **(A, C, E)** The renal tumor. **(B, D, F)** The ureteral tumor.

On admission, ultrasound examination revealed a solid mass at the upper pole of the left kidney. Urinalysis revealed occult blood (2+) and urine leukocytes (+-). Computed tomography (CT) revealed a 56*53 mm soft tissue mass at the upper pole of the left kidney with uneven density and a suspected malignant tumor (**Figures 1A, C**). In addition, a small mass, approximately 1 cm in diameter, was also noted near the upper segment of the right ureter, accompanied by right ureteral dilation and hydronephrosis (**Figures 1B, D**). Computed tomography angiography (CTA) confirmed the left renal mass, showing uneven density (plain scan CT value ~38 HU) with irregular arterial phase enhancement (CT value ~136 HU) and decreased venous phase enhancement (CT value ~92 HU). Tortuous renal arteries were observed traversing the mass, and non-enhancing areas within the mass suggested necrosis. The right ureteral tumor exhibited uneven enhancement, raising suspicion for malignancy.

Given the patient’s bilateral urinary system involvement and advanced age, one-stage surgical management was deemed high risk. Therefore, a staged surgical approach was planned to balance oncological outcomes and preserve renal function after comprehensive consideration. The patient and his family consented to and accepted our proposed treatment plan.

**Figure 2:**
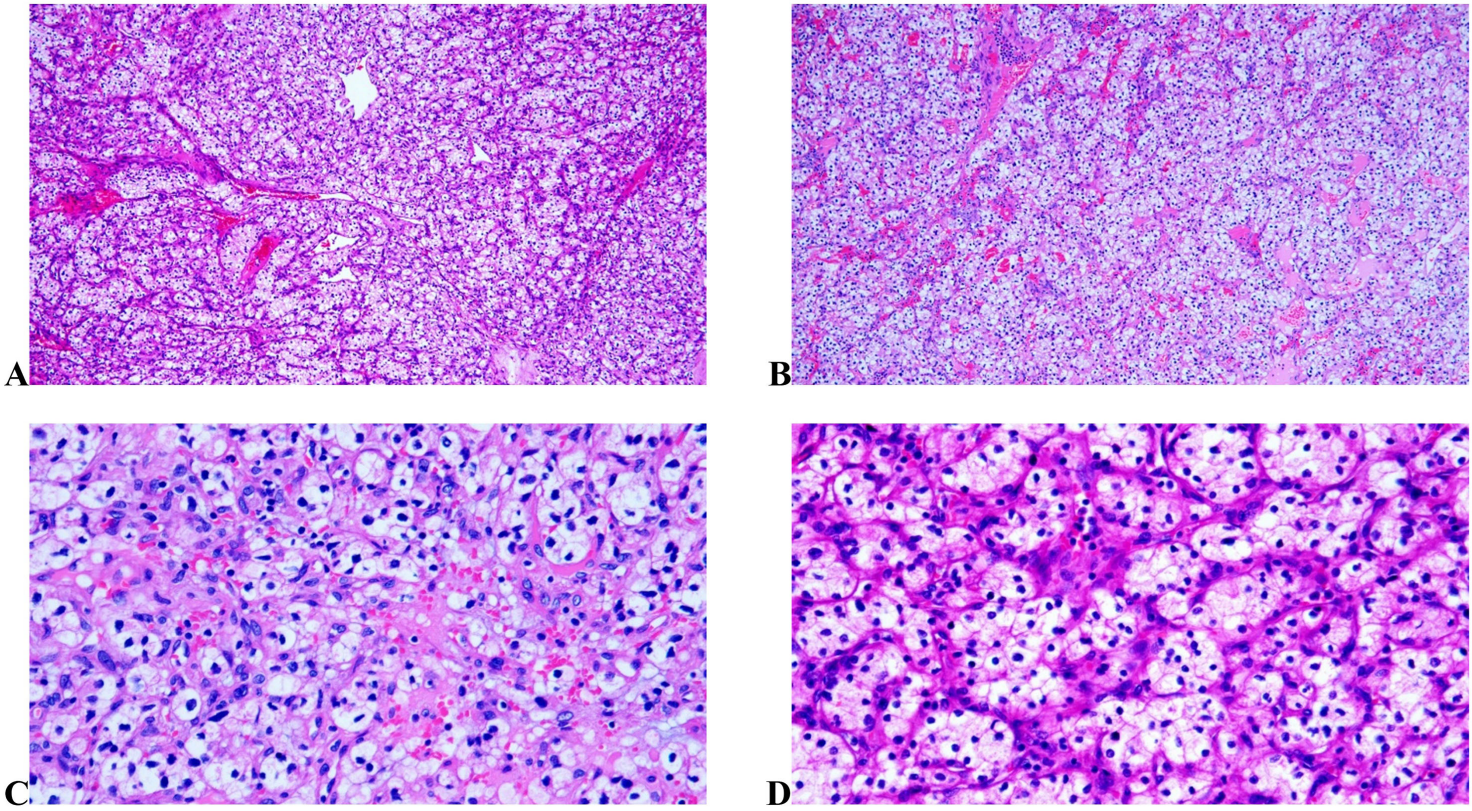
The pathological features under microscope. **(A, B)** The renal carcinoma and the ureteral mass under low magnification, respectively (100×). Under low magnification, the tumor cells are arranged in nests, tubules, or acini, characterized by clear cytoplasm, and the stroma is rich in capillary networks. **(C, D)** The renal carcinoma and the ureteral mass under high magnification, respectively (400×). Under high magnification, the tumor cells appear round or polygonal, large in size, with distinct cell membranes and abundant cytoplasm that is clear, granular or multivacuolated. The nuclei are centrally located and relatively uniform in size.

First, the patient underwent laparoscopic partial nephrectomy for the left renal mass on June 19. Postoperative pathology revealed one tumor measuring approximately 6.55*4 cm in size, with a golden gray–white cut surface and a soft texture. The focal areas are gelatin-like (**Figure 1E**). Microscopically, the tumor was confirmed as clear cell renal cell carcinoma (CCRCC) with focal rhabdoid differentiation, WHO/ISUP grade 4 (**Figures 2A, C**). The immunohistochemistry results were as follows: EMA (+), RCC marker (+), CD10 (+), Vimentin (+), CK7 (-), P504S (focally +), CD117 (-), Ksp-cad (-), TFE3 (focally weak +), CAIX (+), Ki-67 (active area approximately 50% +), and PAX8 (+). Uneventfully, the patient recovered and was discharged on June 30.

Renal dynamic imaging and glomerular filtration rate (GFR) testing prior to the second surgery revealed a total GFR of 62.2 mL/min, with the left kidney at 31.5 mL/min and the right kidney GFR at 30.7 mL/min. The surgery was performed on July 26, involving laparoscopic unilateral nephroureterectomy for the right ureteral tumor. Postoperative pathology revealed a 1.6×1.1 cm pedunculated smooth mass located 14 cm from the ureteral resection margin (**Figure 1F**). Microscopic examination and immunohistochemistry confirmed it as a metastasis of CCRCC, with no cancer involvement at the ureteral resection margin (**Figures 2B, D**).

The patient recovered well and was discharged on August 5. At the fourteen-month follow-up, no evidence of metastasis or other abnormalities was observed. Besides, no special discomfort symptoms have been reported since the last operation.

## Discussions

This is an exceptionally rare case, and we have reviewed the limited number of reported cases reported globally. Typically, the clinical presentation of such cases includes hematuria, as observed in our patient. Early imaging tests, particularly contrast-enhanced CT and urography, play a pivotal role in diagnosis. When a renal tumor is found on one side and a ureteral mass with hydronephrosis on the contralateral side, the possibility of contralateral ureteral metastasis from renal cancer cannot be ignored.

Currently, there is no standardized treatment protocol given the rarity of this condition. Our management strategy is to first address the renal tumor and then address the ureteral side lesion. The optimal treatment for stage T1 renal cancer is partial nephrectomy (9), which can effectively reduce damage to renal function and shorten convalescence. The long-standing standard treatment for upper tract urothelial carcinoma (UTUC) is radical nephroureterectomy, which is the safest method for minimizing the risk of recurrence, although the damage to patients’ renal function is huge. According to the European Association of Urology (EAU) risk stratification tool for UTUC, our case could be classified into the high-risk group, for which radical nephroureterectomy is recommended as the preferred option, as we performed (10). Hence, for this patient, a left partial nephrectomy was performed first to ensure a quicker recovery so that the patient was able to undergo a contralateral radical nephroureterectomy only one month later. However, if we addressed the ureteral lesion first, the operative trauma would have been greater, and the recovery would have been longer, which may have led to the progression of the CCRCC and potentially missed the optimal timing for a partial nephrectomy.

The impact of our surgical approach on renal function is inevitable. However, we have taken concerted measures to minimize this effect. For instance, we maintained close renal function monitoring during follow-up and collaborated with the nephrology department to implement targeted improvements through medication and lifestyle management. Over the recent follow-ups, the patient’s renal function has remained stable, with no observed progressive deterioration. Whereas, the EAU guidelines on UTUC also suggest considering nephron-sparing surgery for patients with a solitary kidney and/or impaired renal function, which is noteworthy for patients with RCC with contralateral ureteral metastasis. In our case, the patient’s renal function was impaired after partial nephrectomy. Thus, whether nephron-sparing surgery on the contralateral side can be an option is discussable. If this surgery was to be performed, then cytological examination is necessary, such as preoperative ureteroscopic biopsy or rapid intraoperative pathology, which contributes to clarifying the tumor’s pathological characteristics and determining its risk accurately though it should be noticed that ureteroscopic biopsy for UTUC might increase the risk of intravesical recurrence (11). Other limitations of ureteroscopic biopsy include possible missed diagnoses or inaccurate staging due to insufficient sampling (12, 13), as well as the risk of tumor dissemination caused by high intraluminal pressure, mucosal injury, or ureteral perforation during the procedure (14, 15). Owing to the scarcity of similar cases and the lack of long-term follow-up, the safety of nephron-sparing surgery alone remains to be verified.

One noteworthy aspect in our case is the pathological finding. While no mention of rhabdoid differentiation was found in the reported similar cases, this differentiation occurs in 4–6% of RCC overall, and its frequency increases to over 27% in high-grade (WHO/ISUP grade 4) RCCs (16–18). This morphological pattern is linked to early metastasis and poor prognosis (17, 19, 20). Although less aggressive than sarcomatoid differentiation (21), its presence in our case likely contributed to the contralateral ureteral metastasis. While VHL inactivation is the primary driver of clear cell renal cell carcinoma, our study did not include in-depth research like genetic analysis of the tumor. This represents one of the limitations of our study. Current evidence shows that rhabdoid differentiation often involves additional high-grade mutations, such as co-occurring BAP1 and PBRM1 alterations (22). Therefore, conducting fundamental researches in such cases would be valuable for understanding tumor pathogenesis and investigating potential targets for treatment. Hence, we propose creating a multi-institutional registry to systematically identify and manage these rare cases. This initiative will not only create opportunities for fundamental research but also facilitate the collection of comprehensive clinical data, ultimately enhancing our understanding of the disease’s pathogenesis and refining clinical management strategies.

## Conclusions

We report a case of postoperatively confirmed unilateral renal clear cell carcinoma with synchronous contralateral ureteral metastasis, which is an extremely rare occurrence. Our treatment approach involved firstly management of the renal tumor, followed by the contralateral ureteral tumor. Although the huge damage to the patient’s renal function is inevitable, the advantage of this approach is that it ensures optimal safety and minimizes the interval between surgeries, thus preventing progression of the disease.

## Data Availability

The original contributions presented in the study are included in the article/Supplementary Material. Further inquiries can be directed to the corresponding author.
